# A Longitudinal Comparison of the Recovery Patterns of Optic Neuritis with MOG Antibody-Seropositive and AQP4 Antibody-Seropositive or -Seronegative for Both Antibodies

**DOI:** 10.1155/2022/4951491

**Published:** 2022-03-22

**Authors:** Lin Zhou, Xiao Tan, Ling Wang, Xiujuan Zhao, Wei Qiu, Hui Yang

**Affiliations:** ^1^Department of Ophthalmology, West China Hospital, Sichuan University, Chengdu 610041, China; ^2^Shenzhen Aier Eye Hospital Affiliated to Jinan University, Shenzhen, Guangdong 518032, China; ^3^Department of Medical Retina and Neuro-Ophthalmology, Zhongshan Ophthalmic Center, Sun Yat-Sen University, Guangzhou 510060, China; ^4^Department of Neurology, The Third Affiliated Hospital of Sun Yat-Sen University, Guangzhou 510060, China

## Abstract

In this study, the aim is to compare the recovery pattern among patients with acute myelin oligodendrocyte glycoprotein antibody-seropositive optic neuritis (MOG-Ab + ON) attacks and aquaporin-4 antibody-seropositive ON (AQP4-Ab + ON) or -seronegative ON. At the onset of the first-ever ON attack, the thickness of RNFL (RNFLt) in the MOG-Ab + ON group was significantly thicker than others (*P* < 0.05), while visual function damage was not significantly different to other groups. One month to six months after onset, the MOG-Ab + ON group showed significantly better visual function (*P* < 0.05) than the other two groups, while the RNFLt showed no significant difference among the three groups (*P* > 0.05). MOG-Ab + ON and AQP4-Ab + ON groups showed rapid recovery in the first month and then plateaued. The annual relapse rate was significantly higher in MOG-Ab + ON and AQP4-Ab + ON groups than seronegative ON. The relapse interval of the MOG-Ab + ON group (9.00 ± 7.86 months) was significantly shorter than that of the AQP4-Ab + ON group (45.76 ± 37.82 months) (*P* < 0.05) but showed no significant difference from that of the seronegative ON group (*P* > 0.05). To sum up, the recovery patterns were different among these three types of ON. RNFLt was not parallel to the recovery of visual function among these types of ON. MOG-Ab + ON had the mildest visual function damage but the most substantial RNFL changes, while AQP4-Ab + ON suffered the worst function damage. MOG-Ab + ON had a similar relapse rate as AQP4-Ab + ON but a shorter interval, indicating that relapse prevention was necessary and should be initiated as early as possible.

## 1. Introduction

Optic neuritis (ON), defined as inflammation of the optic nerve, results in various degrees of visual function damage heralded or accompanied by retro-orbital and/or eye movement pain [1]. If patients are not treated promptly and properly, they may develop permanent vision loss [2]. ON can occur in isolation, as a recurrent condition, or as the initial symptom of a central nervous system (CNS) demyelinating disease, such as multiple sclerosis (MS) or neuromyelitis optica spectrum disorder (NMOSD).

Recently, MOG-IgG-associated demyelinating disease (MOGAD) was recognized as a new disease entity, different from MS and AQP4-Ab-positive NMOSD [3, 4]. Optic neuritis was the most frequent phenotype of MOGAD. This subset of ON (MOG-Ab + ON) has some clinical characteristics that differentiate it from other types of ON. MOG-Ab + ON has a much higher relapse rate and a much shorter relapse interval than other types of ON, but the annual relapse rate is lower in pediatric MOG-Ab + ON than in adults [5, 6]. Magnetic resonance imaging (MRI) showed involvement of the long segments and preferentially the anterior segments of the optic nerve, resulting in a high incidence of optic disc swelling. The intracanalicular segment, optic chiasm, and optic tract are less involved in this condition than in NMOSD-ON. Orbital connective tissue involvement on MRI is unique to MOG-Ab + ON compared to other types of ON [7–11]. For patients with timely treatment, the prognosis was found to be better than for other types of ON. However, latent atrophy of the optic nerve could be detected in patients with MOG-Ab + ON, which could also be found in MS-ON [12, 13].

However, there are still some unclear clinical features of MOG-Ab + ON. Most of the clinical studies about MOG-Ab + ON were about a certain aspect at a certain stage of the disease, such as the BCVA or RNFLt alone at 6 months after onset [14–17]. To the best of our knowledge, few longitudinal studies on the recovery pattern during an acute attack of MOG-Ab + ON have been conducted. In this study, we aim to illustrate the longitude recovery pattern of MOG-Ab + ON by comparing it with AQP4-Ab + ON and seronegative ON from acute onset until six months after, both at the first-ever ON attack and the relapsed ON.

## 2. Materials and Methods

### 2.1. Subjects

This was a prospective, single-center, observational study. It has been approved by the institutional review board of Zhongshan Ophthalmic Center of Sun Yat-Sen University. Written informed consent in accordance with the tenets of the Declaration of Helsinki was obtained from patients or the guardians of the children before all of the clinical data and blood samples were collected. Consecutive patients with acute ON attacks were recruited from the Zhongshan Ophthalmic Center from November 2019 to April 2021. The patients were diagnosed with ON based on the optic neuritis treatment trial guidelines. Patients with autoimmune-related diseases, infectious ON, and other types of ON and patients with unavailable MOG-Ab or AQP4-Ab testing were excluded. Serum samples were obtained from recruited patients within 1 month (M) after the ON attack. A cell-based assay method was used for the detection of AQP4-Ab and MOG-Ab for all recruited patients. In our study, both eyes in one patient could be recruited. Regardless of the left or right eye, once one of the eyes had been attacked, it would be included in the analysis separately.

### 2.2. Treatment Strategy

For the patients with first-ever attack in MOG-Ab + ON, they received 1,000 mg per day of intravenous methylprednisolone (IVMP) for 3–5 days. Plasmapheresis (PLEX) or intravenous immunoglobulin (IVIG) could be considered especially if the patient had severe vision loss and did not show demonstrable improvement after the treatment for 1-2 weeks. A 3-month prednisone taper is adapted. If high levels of MOG-Ab + persisted, maintenance prednisone could be used for 6 months. If patients had not fully recovered or had steroid dependence, treatment to prevent recurrence (mycophenolate mofetil (MMF) or IVIG every three weeks) was necessary. For the patients with recurrent attack in MOG-Ab + ON, 1,000 mg per day of IVMP for 3–5 days is used. 6 months of prednisone tapering was followed by relapse prevention (MMF or IVIG every 3 weeks) (Figure S1). Patients with seronegative ON received methylprednisolone 1 g for three days and then 1-2 mg/kg prednisone for 11 days and then gradually tapered within 3 months. Patients with AQP4-Ab + ON received methylprednisolone 1 g for three days, 0.5 g for three days, 0.25 g for three days, and 120 mg for three days and then 1-2 mg/kg prednisone for 11 days and gradually tapered. Prednisone 5 mg per day and azathioprine 50 mg twice a day were used as a long-term preventive treatment for patients with AQP4-Ab + ON.

### 2.3. Clinical Data Collection

A comprehensive medical record history and ocular examinations were conducted and collected at onset (within one week of the onset of symptoms), 1 M, 3 M, and 6 M after attack. The ocular examinations included the best corrected visual acuity (BCVA), visual field (VF), visual evoked potentials (VEPs), and optical coherence tomography (OCT). The BCVA was converted to the logarithm of the minimum angle of resolution (LogMAR). A comparison of the clinical data of the first-ever ON attack and after multiple attack was conducted within each group at onset, 1 M, 3 M, and 6 M after attack.

VF sensitivity was assessed by the central 30-2 SITA program on a Humphrey Visual Analyzer (Carl Zeiss Meditec, Dublin, California). If the false-positive or -negative rate was greater than 15% or fixation loss was more than 33%, the examination should be repeated until the quality had achieved the standard. The mean deviation (MD) was used to record the overall visual field damage. If the BCVA was worse than 0.01, the MD would be defined as −34 dB. Pattern VEP recordings (Espion, Diagnosys, America) were performed following the guidelines of the International Society for Clinical Electrophysiology of Vision. Pattern reversal VEPs used a black and white checkerboard at a reversing frequency of 2 Hz, with full-field stimulation, a contrast level of 100%, a parsing time of 250 ms, and an average time of 200 ms. The latency and amplitude of the P100 component for a stimulus size of 60 min arc were analyzed. Abnormal data were defined as exceeding 2 SD from the normal range according to data from healthy controls of the same age and gender. When no wave could be detected, the amplitude was recorded as 0 and latency was regarded as unidentified. The thickness of the retinal nerve fiber layer (RNFL) of the optic nerve, with measurements centered at the optic disc with a diameter of 3.4 mm, was acquired by OCT (Heidelberg Engineering, Heidelberg, Germany). The overall average thickness of the RNFL and the RNFL thickness of each quadrant, including the superior, inferior, temporal, and nasal areas, were analyzed.

### 2.4. Statistical Analysis

Statistical analysis was performed using STATA 23.0 software. Student's *t*-test and *t*-test with Bonferroni correction were used for parametric comparisons of the clinical parameters. The Kruskal–Wallis test was used for nonparametric comparisons. *R* × *C* table chi-squared tests or Fisher's exact tests were used for the comparison of the categorical data. Statistical significance was defined as a 2-sided *P* value less than 0.05.

## 3. Results

A total of 136 consecutive patients with acute ON were recruited for this study, and only 113 patients finished all the 6 M-follow-up and required examinations, including 35 (31.0%) patients with MOG-Ab + ON, 38 (33.6%) patients with AQP4-Ab + ON, and 38 (35.4%) patients with seronegative ON. Two (1.8%) patients were seropositive for both MOG-Ab and AQP4-Ab (Table 1). Since these double-positive patients are rare and were not encountered in large enough numbers to conduct a statistical analysis, they were not included in the study.

Among the 113 ON patients recruited, 55.8% (63/113) had a first-ever ON attack, including 37.1% (13/35) patients with MOG-Ab + ON, 31.6% (12/38) patients with AQP4-Ab-ON, and 95.0% (38/40) patients with seronegative ON. Additionally, 44.2% of patients suffered the relapse of ON. We will conduct a comprehensive longitude comparison among these three groups.

**Figure 1:**
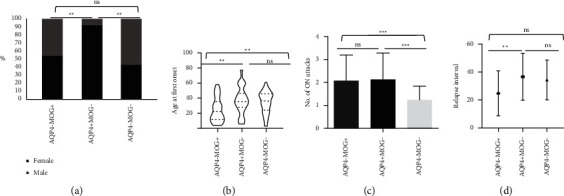
The basic clinical data of the patients at the first-ever ON attack. (a) The comparison of gender among the patients with MOG-Ab + ON, AQP4-Ab + ON and AQP4-MOG-ON. (b) The comparison of age at first-ever ON attack. (c) The comparison of no. of ON attacks. (d) The comparison of relapse interval between the first attack and the second attack.

The demographic and clinical characteristics of all recruited patients are listed in Table 1. No significant difference in gender was found between the MOG-Ab + ON group and the seronegative ON group, but a significant female predominance was detected in patients with AQP4-Ab + ON (*P* < 0.001) (Figure 1(a)). The age of first attack for patients with MOG-Ab + ON was significantly younger than patients with AQP4-Ab + ON (*P* < 0.01) and seronegative ON (*P* < 0.01). However, we did not find difference in age between patients with AQP4-Ab + ON and seronegative ON (*P* > 0.05) (Figure 1(b)). The time interval between the first attack and the second ON attack was significantly shorter in MOG-Ab + ON group (9.00 ± 7.86 months) than in the AQP4-Ab + ON group (45.77 ± 37.82 months) (*P* < 0.01). Though the time interval of the AQP4-Ab + ON group was also shorter than the seronegative ON group (24.00 ± 27.98 months), no significant difference was detected (*P* > 0.05) (Figure 1(c)). The relapse rate was significantly lower in the seronegative group (1.25 ± 0.58) than in the other two groups (*P* < 0.05), while no significant difference was found between the AQP4-Ab + ON (2.13 ± 1.15) and MOG-Ab + ON (2.08 ± 1.11) groups (Figure 1(d)).

### 3.1. Analysis of Patients with MOG-Ab + ON

#### 3.1.1. The First-Ever Attack of MOG-Ab + ON

**Figure 2:**
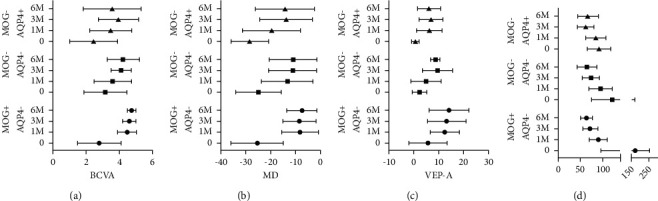
The longitude comparison of clinical data among patients with MOG- + AQP4-ON, AQP4-Ab + ON and AQP4-MOG-ON at first-ever ON attack. (a) The comparison of the best corrected visual acuity. (b) The comparison of the mean deviation of visual field. (c) The comparison of amplitude of visual evoked potential (VEP). (d) The comparison of average of the thickness of retinal nerve fiber layer. The visual function including BCVA, MD, and the amplitude of VEP of the MOG-Ab + ON is the best among the three groups throughout the course of the disease at the first onset. Patients with MOG-Ab + ON and AQP4-Ab + ON showed a rapid recovery at 1 M and then plateaued at 1 M to 6 M after the onset of the first-ever attack. However, the recovery of patients with seronegative ON was gradually increased. (d) Significant swelling of the retinal nerve fiber layer could be detected in patients with MOG-Ab + ON. However, there was no difference in the thickness of the retinal nerve fiber layer among the three groups after 6 M. The thickness of the retinal nerve fiber layer of three groups all gradually thinning and shrinking during the course at the first onset.

For the MOG-Ab + ON group, BCVA (2.80 ± 1.32 at onset vs 4.47 ± 0.58 at 1 M, *P* < 0.0001), MD (−25.43 ± 10.47 at onset vs −8.28 ± 7.40 at 1 M, *P* < 0.0001), and the amplitude of VEP (5.63 ± 7.67 at onset vs 12.41 ± 5.87 at 1 m, *P* < 0.001) showed significantly improved at 1 M after onset (Table 1 and Figure 2(a)). However, no significant differences among the 1 M, 3 M, and 6 M groups were found, which means that patients with MOG-Ab + ON showed rapid increases in BCVA, MD, and VEP-A within 1 M and then leveled off during 1 M to 6 M (Figure 2). Significant clinically recorded swollen RNFL (175.00 ± 78.48) was detected at the onset of ON, which meant the swollen area was visible to the naked eye. The RNFLt in MOG-Ab + ON is significantly thicker than in other two groups (*P* < 0.05) at onset. While 1 M after onset, the RNFL of MOG-Ab + ON (90.08 ± 19.74) became significantly thinner comparing with the onset data (*P* < 0.05) and then leveled off. No significant differences among the RNFLt at 1 M, 3 M, and 6 M were found (Figure 2(d)).

#### 3.1.2. Relapsed MOG-Ab + ON Group

**Figure 3:**
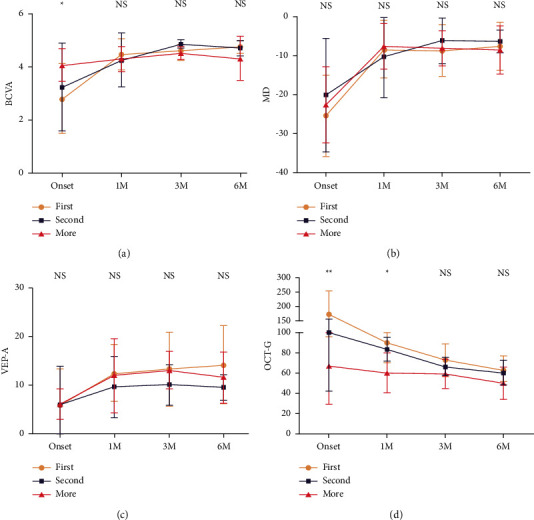
The comparison of the clinical data for patients with MOG-Ab + ON among the first attack, the second attack, and multiple attacks. (a) The comparison of the best corrected visual acuity. (b) The comparison of the mean deviation of visual field. (c) The comparison of amplitude of visual evoked potential (VEP). (d) The comparison of average of the thickness of retinal nerve fiber layer. (a–c) The visual function was not significantly decreased after twice or more attacks compared with the first attack. (d) The RNFL became thinner with each ON attack, though no significant difference could be found. The tendency of the change of RNFL of the optic nerve could be more and more stead and gentle with each attack.

Among all 35 patients with MOG-Ab + ON, 22 patients had relapsed ON. Among them, 12 patients suffered from a second ON attack and 10 suffered from a third ON attack. Regarding visual function, including BCVA, MD, and VEP-A, no significant difference was found between the first-ever ON group and the relapsed ON group. A trend of RNFL thinning could be found with each ON attack without a significant difference (*P* > 0.05) (Figure 3). No significant difference in RNFL thickness was found among different quadrants (Figure S2).

### 3.2. Analysis of Patients with AQP4-Ab + ON

#### 3.2.1. The First-Ever Attack of Patients with AQP4-Ab + ON

The recovery trend of visual function, including BCVA (2.46 ± 1.45 vs 3.47 ± 1.28, *P* < 0.0001), MD (−28.47 ± 7.43 vs −19.74 ± 11.63, *P* < 0.0001), and VEP-A (0.61 ± 1.47 vs 6.2 ± 5.09, *P* < 0.001), is similar to MOG+, which showed rapid recovery within the first month and then leveled off (Figure 2). The RNFLt of the optic nerve gradually decreased, and a significant difference was found between the onset and 3 M (91.91 ± 25.94 vs 61.92 ± 19.26, *P* < 0.05) and between the onset and 6 M (91.91 ± 25.94 vs 67.43 ± 22.86, *P* < 0.05). However, it could be leveled off during 3 M and 6 M (Figure 2(d)).

#### 3.2.2. Relapsed Attack of Patients with AQP4-Ab + ON

**Figure 4:**
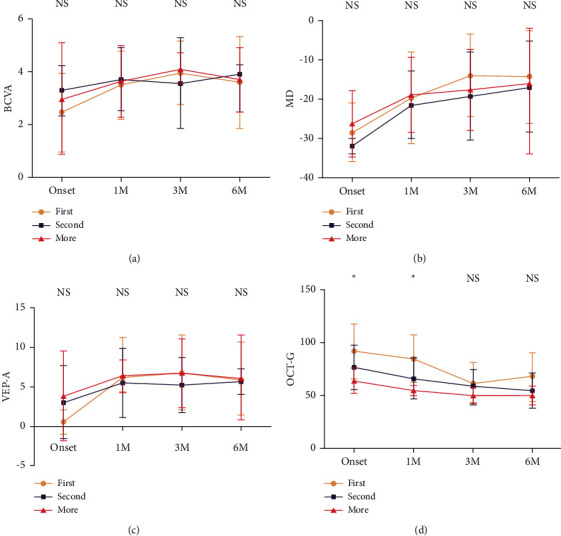
The comparison of the clinical data for patients with AQP4-Ab + ON among the first attack, the second attack, and multiple attacks. (a) The comparison of the best corrected visual acuity. (b) The comparison of the mean deviation of visual field. (c) The comparison of amplitude of visual evoked potential (VEP). (d) The comparison of average of the thickness of retinal nerve fiber layer. (a–c) Significant difference could not detect among the first attack, the second attack, and multiple attacks. (d) While gradually thinning of the RNFL could be detected with each ON attack, though no significant difference could be found.

A total 26 patients (68.4%) suffered the relapsed ON, including 12 patients from a second ON attack and 14 from a third ON attack. The recovery trend of AQP4-Ab + ON is similar to that of patients with MOG-Ab + ON. Significant differences could not be detected among the first attack and the second attack and after multiple attacks. While gradually thinning of the RNFL could be detected with each ON attack, no significant difference in RNFL thickness was found among different quadrants. The changes in the thickness of the RNFL of the optic nerve are identical among the superior, inferior, nasal, and temporal regions of the optic nerve (Figure 4 and Figure S1).

### 3.3. Analysis of Patients with Seronegative ON

#### 3.3.1. The First-Ever Attack of Patients with Seronegative ON

The visual function and structure were similar to the other two groups. The recovery of BCVA and VEP-A and MD gradually increased, and a significant difference could be found between onset and 6 M, between onset and 3 M (Figure 2). There were no differences detected between onset and 1 M, between 1 M and 3 M, and between 3 M and 6 M. The RNFLt of the optic nerve gradually decreased, and a significant difference was found between onset and 3 M (122.79 ± 46.96 vs 74.29 ± 18.78, *P* < 0.05) and between onset and 6 M (122.79 ± 46.96 vs 65.2 ± 21.88, *P* < 0.05). However, it could be leveled off during 3 M and 6 M (Figure 2(d)).

#### 3.3.2. Relapsed Attack of Patients with Seronegative Group

A total of 8 patients suffered relapsed ON. Only six patients suffered two attacks, and two patients suffered more than two attacks in the seronegative group. Due to the limited sample size, we did not conduct a comparison among several attacks.

### 3.4. Comparison of the Recovery Trend at the First Attack among Three Different Groups

The visual function of patients with MOG-Ab + ON and AQP4-Ab + ON rapidly improved during the first month and then leveled off, while seronegative patients recovered gradually over the 6 M after onset (Figure 2(a)). Significant differences could be detected among the three months during the 6 M after onset. The visual function of MOG-Ab + ON is the best recovered, and AQP4-Ab + ON was the worst. Only patients with MOG-Ab ON + suffered swelling of the optic nerve at the onset of the first-ever attack. However, the thickness of RNFL is similarly damaged among MOG-Ab + ON, and there was no significant difference in the thickness of the RNFL among the three groups after the onset of the first-ever attack (Figure 2(d)).

### 3.5. Comparison of the Recovery Trend after Multiple Attacks between Patients with MOG-Ab + ON and Patients with AQP4-Ab + ON

**Figure 5:**
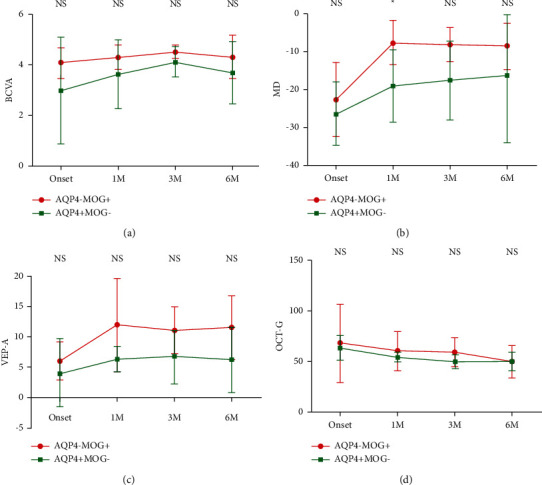
Comparison of the recovery trend after multiple attacks between patients with MOG-Ab + ON and patients with AQP4-Ab + ON. (a) The comparison of the best corrected visual acuity. (b) The comparison of the mean deviation of visual field. (c) The comparison of amplitude of visual evoked potential (VEP). (d) The comparison of average of the thickness of retinal nerve fiber layer. The visual function (BCVA, MD, and VEP) of patients with MOG-Ab + ON is better than that of patients with AQP4-Ab + ON from the onset to 6 M during the course of the last attack, although no significance could be detected. The impairment of the thickness of the RNFL of the optic nerve is similar to each other.

The visual function (BCVA, MD, and VEP) of patients with MOG-Ab + ON is better than that of patients with AQP4-Ab + ON from onset to 6 M during the course of the last attack, although no significance could be detected (Figure 5). The impairment of the thickness of the RNFL of the optic nerve is similar to each other (Figure 5). The thickness of the RNFL might be good indicator for the structural damage to the MOG-Ab-ON.

## 4. Discussion

To the best of our knowledge, our study is the first longitudinal, prospective, and comprehensive visual functional and structural comparison among MOG-Ab + ON, AQP4-Ab + ON, and seronegative ON at first-ever attack and after multiple attacks. Without the influence of previous attacks, the acute phase of ON (from onset to 6 months after onset) of first-ever ON is the best window for the observation of recovery patterns. Our results illustrated that the impairment style, the trend of recovery, and the prognosis were all quite different among these three types of ON, with special implications for the diagnosis, treatment, and prevention.

Currently, there is much controversy regarding the treatment of MOG-Ab + ON [18–20]. Some suggested that we should start with steroid pulse therapy using the regimen for anti-AQP4 antibody positive ON, followed by posttherapy administration of oral steroids (plus immunotherapy) [21–23]. Our results show that the current steroid regimen identical to that for seronegative ON is enough for satisfactory effect for the acute stage. But it is not clear whether a more aggressive steroid regimen might be helpful for prevention of relapse and worthwhile for the higher risk of steroid-related side effects that comes with it. In this study, in regard to the recovery patterns during the acute phase of the first-ever ON attack, MOG-Ab + ON and AQP4-Ab + ON achieved the most prompt recovery within the first month and then plateaued. However, seronegative ON gradually recovered until 6 M after onset. Although swift administration of steroid pulse therapy is desirable in the acute phase of all ON, the recovery trend results in this study further suggest that the clinical results at 1 M after onset might be an index for prognosis of MOG-Ab + ON and AQP4-Ab + ON. As for seronegative ON, the recovery process continued to 6 M after onset, which might indicate a prolonged steroid therapy regimen different from that suggested by the ONTT [24, 25], which started with three days of impulse steroid therapy, reduced the dose to 1-2 mg/kg, and then rapidly tapered it to withdrawal within two weeks. Although MOG-Ab + ON and seronegative ON shared the same therapy regimen, their recovery trends were different. The result suggested that if patient showed significant swollen disc with prompt satisfactory to the steroid treatment alone at 1 M after onset and then plateau, a disposition to diagnosis of MOG-Ab + ON might be considered.

The relapse-preventive therapies for patients with MOG-Ab + ON in the chronic phase have not yet been established. Although steroid therapy is effective for the acute phase of MOG-Ab + ON, frequent and rapid relapses and the development of other CNS lesions remain as issues. In such cases, MOG-Ab + ON could be considered a refractory disease [20], and prevention of relapse is a very important issue. The number of relapses of ON attack in patients with MOG-Ab + varied from once to 4.5 times, while that of patients with AQP4-Ab + varied from 1.33 to 4.72 times in the literature [13–17, 22, 24, 26–58]. The results from the literature are conflicting; some reports state that MOG-Ab + ON is more likely to relapse [37, 59], and some reports state that AQP4-Ab + ON disease is more likely to relapse [15]. The higher relapse rate in MOG-Ab + ON and AQP4-Ab + ON might be due to established B cells producing specific MOG and AQP4 Ab. These B cells might be easily stimulated by changes in the immunological environment. Therefore, immunosuppressant therapy is needed to prevent relapse. In this study, the time of relapse was not significantly different between the MOG-Ab + ON and AQP4-Ab + ON groups. The time interval before relapse in MOG-Ab + ON (9.00 ± 7.86 months) was significantly shorter than those in AQP4-Ab + ON (45.77 ± 37.82 months) and seronegative ON (24 ± 27.98 months) groups. These results might indicate that relapse prevention for patients with MOG-Ab + ON was necessary and should be initiated as early as possible because of the rapid recurrence. Regarding the exact regimen for prevention, there was no high-quality evidence from clinical research. Immunosuppressant agents, including low-dose oral prednisolone as adopted in AQP4-ON [20, 60, 61], might be used. Disease-modifying drugs used for relapse prevention in MS-ON should be avoided. It is now clear that AQP4-Ab + ON is an astrocyte disease with robust axon damage in severe cases, and demyelination is only a secondary change [62]. The main pathological change in patients with MOG-Ab + ON was not yet clear, and the pathological damage might be restricted to myelin, with the axons relatively preserved. The only direct evidence was from the brain biopsies of two patients with MOG-associated demyelinating pseudotumors. The histological features included T cells, macrophages, and complement-mediated demyelination, which was similar to that of MS [23]. The swollen of the RNFL in patients with MOG-Ab + ON was most significant, while the visual function damage was the mildest among these three groups after onset, especially at 6 M after onset, indicating that the main pathological change in MOG-Ab + ON might be largely restricted to the myelin of the optic head, while the axon was relatively waived. Recent reports of recessive optic atrophy in MOG-Ab + ON, i.e., no changes in visual function, such as visual field, but thinning of RNFL, further support this hypothesis [13, 62, 63]. Although, the RNFLt had no significant difference among the three group as early as 1 m after onset, the visual function was significantly different with AQP4-Ab + ON had the worst visual function damage and MOG-Ab ON group had least damage. This indicated that RNFLt was not paralleled to the visual function damage. In this case, whether GCL be a more sensitive and appropriate index for detecting optic nerve damage remained to be further investigated.

It has been suggested that MOG-Ab + ON is a mild and usually multiphasic variant of ON, while MOG + NMOSD-ON might be a severe variant of NMOSD. In AQP4-Ab-negative NMO patients, approximately 12–20% of patients were MOG+ and were therefore defined as MOG + NMOSD-ON [49, 50, 64]. In this study, all three groups achieved a significant recovery in visual function after six months of follow-up. Patients with MOG-Ab + ON achieved faster and better recovery than the other two groups in the first 6 M after the ON attack. This outcome was quite different from what was reported for MOG-Ab + NMOSD-ON, which was reported to be much more severe than AQP4-Ab + ON and seronegative ON. This distinction indicated that the pathogenesis of these two types of ON might be quite different and that the two types might be different entities, although both are MOG-Ab seropositive.

However, there are some limitations in this study. (1) the sample size in our study is insufficient. A small sample size may make it difficult to determine if a particular outcome is a true finding. In future research, we should collect more samples for further analysis and comparison. (2) The follow-up time is too short. We cannot fully record the prognosis of patients with more than 6 m and later recurrence. Further study is needed to analysis the prognosis of the MOG-Ab + ON and AQP4-Ab + ON after 6 M.

## 5. Conclusions

The recovery patterns were different among these three types of ON: MOG-Ab + ON, and AQP4-Ab + ON mostly recovered within the first month and then plateaued. However, seronegative ON patients gradually recovered till 6 M after the onset. MOG-Ab + ON had the mildest visual function damage but the most substantial RNFL changes, indicating that the main pathological change in MOG-Ab + ON might be largely restricted to the myelin of the optic head. MOG-Ab + ON had a similar relapse rate as AQP4-Ab + ON but a shorter interval, indicating that relapse prevention was necessary and should be initiated as early as possible.

## Figures and Tables

**Table 1:** Comparison of the basic information among the patients with AQP4-Ab + ON, MOG-Ab + ON, and seronegative ON at first ON onset.

	MOG-Ab + ON	AQP4-Ab + ON	Seronegative ON	Among three groups	*M*+ vs *A*+	*M*+ vs —	*A*+ vs —
Basic clinical data							
Number of patients and eyes	24, 30	36, 43	40, 40				
Female, *n* (%)	15, 50%	39, 91%	17, 43%	^*∗∗∗*^	^*∗∗∗*^	ns	^*∗∗∗*^
Relapse interval	9.00 ± 7.86	45.77 ± 37.82	24 ± 27.98	^*∗*^	^*∗∗*^	ns	ns
Age at first onset	24.83 ± 15.79	36.67 ± 16.55	34.45 ± 13.96	^*∗∗*^	^*∗∗*^	^*∗∗*^	ns
No. of ON attacks	2.08 ± 1.115	2.13 ± 1.15	1.25 ± 0.58	^*∗∗∗*^	ns	^*∗∗∗*^	^*∗∗∗*^
At the first onset							
VA, logMar	2.80 ± 1.32	2.46 ± 1.45	3.17 ± 1.30	ns	ns	ns	ns
MD of visual field	−25.43 ± 10.47	−28.47 ± 7.43	−25.07 ± 9.08	ns	ns	ns	ns
RNFL	175.00 ± 78.48	96.30 ± 22.97	122.79 ± 46.96	^*∗∗*^	^*∗∗*^	ns	^*∗*^
The first onset after 1 M							
VA, logMar	4.48 ± 0.58	3.49 ± 1.28	3.61 ± 1.12	^*∗∗*^	^*∗∗*^	^*∗∗*^	ns
MD of visual field	−8.28 ± 7.40	−19.74 ± 11.63	−13.43 ± 10.33	^*∗∗*^	^*∗∗*^	ns	ns
RNFL	90.08 ± 19.74	84.71 ± 22.87	95.33 ± 26.10	ns	ns	ns	ns
The first onset after 3 M							
VA, logMar	4.62 ± 0.40	3.95 ± 1.22	4.12 ± 0.60	^*∗*^	^*∗*^	^*∗*^	ns
MD of visual field	−8.61 ± 6.67	−13.91 ± 10.51	−11.29 ± 9.66	ns	ns	ns	ns
RNFL	72.75 ± 16.47	61.92 ± 19.26	74.29 ± 18.78	ns	ns	ns	ns
The first onset after 6 M							
VA, logMar	4.75 ± 0.25	3.58 ± 1.74	4.24 ± 0.97	^*∗*^	^*∗*^	ns	ns
MD of visual field	−7.51 ± 6.07	−14.36 ± 11.81	−11.08 ± 9.52	ns	ns	ns	ns
RNFL	64.08 ± 13.11	67.43 ± 22.86	65.20 ± 21.88	ns	ns	ns	ns

Note: *N*: number; *F*: female; *M*: male; *M*+ vs *A*+: MOG-Ab + ON vs AQP4-Ab + ON; *M*+ vs —:MOG-Ab + ON vs seronegative ON; *A*+ vs —: AQP4-Ab + ON vs seronegative ON.

## Data Availability

The data used to support the findings of this study are available from the corresponding author upon request.

## Supplementary Materials

Supplementary Materials Figure S1: a flow chart for the treatment of MOG-Ab + ON. (a) The flow chart for the treatment of first-ever attack in MOG-Ab + ON. (b) The flow chart for the treatment of recurrent attacks in MOG-Ab + ON. Figure S2: the comparison of the thickness of the retinal nerve fiber layer of the patients. (a) The comparison thickness of retinal nerve fiber layer at the first-ever attack among different quadrants (center, superior, nasal, inferior, and temporal) in patients with MOG + AQP4-ON. (b) The comparisons of thickness of the retinal nerve fiber layer among patients with MOG + AQP4-ON and MOG-AQP4 + ON at different attacks. (c) The comparisons of thickness of the retinal nerve fiber layer of the patients with AQP4-Ab + ON at different attacks.
