# Supplementary figures and images for: A Longitudinal Comparison of the Recovery Patterns of Optic Neuritis with MOG Antibody-Seropositive and AQP4 Antibody-Seropositive or -Seronegative for Both Antibodies
Source: J Ophthalmol. 2022 Mar 22;2022:4951491. doi: 10.1155/2022/4951491 (PMC8964224; doi:10.1155/2022/4951491)

**A**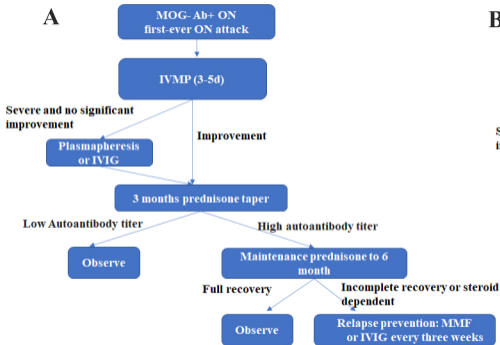**B**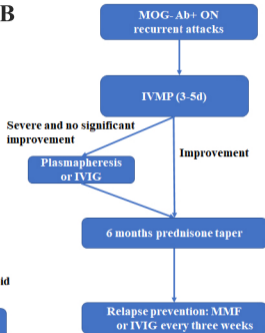

Supplement: Supplementary Materials — Figure S1: a flow chart for the treatment of MOG-Ab + ON. (a) The flow chart for the treatment of first-ever attack in MOG-Ab + ON. (b) The flow chart for the treatment of recurrent attacks in MOG-Ab + ON. Figure S2: the comparison of the thickness of the retinal nerve fiber layer of the patients. (a) The comparison thickness of retinal nerve fiber layer at the first-ever attack among different quadrants (center, superior, nasal, inferior, and temporal) in patients with MOG + AQP4-ON. (b) The comparisons of thickness of the retinal nerve fiber layer among patients with MOG + AQP4-ON and MOG-AQP4 + ON at different attacks. (c) The comparisons of thickness of the retinal nerve fiber layer of the patients with AQP4-Ab + ON at different attacks. [file 4951491.f1.zip › 4951491.f1/Figure S1.pdf]

Figure S.2: The comparisons of thickness of the retinal nerve fiber layer of the patients.

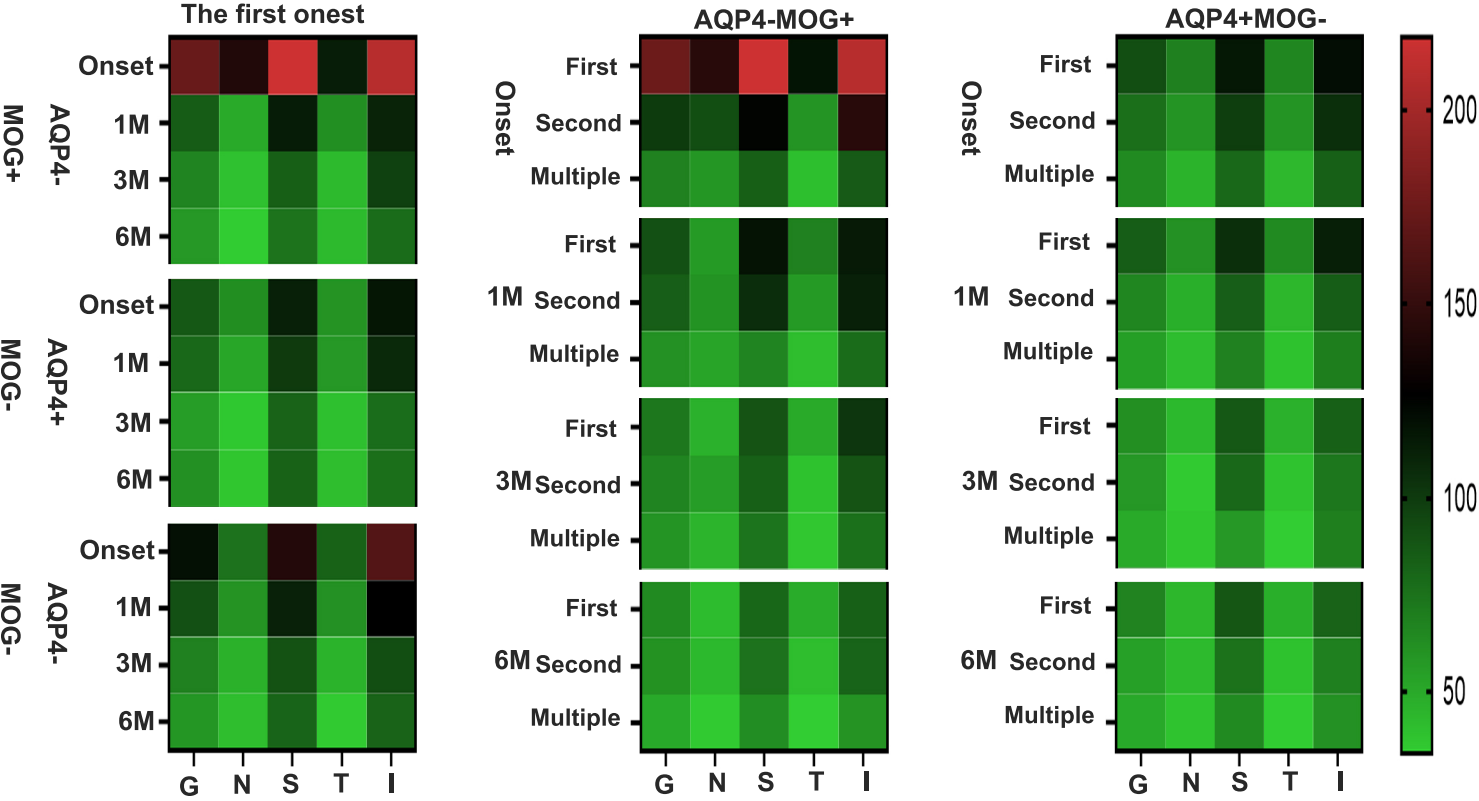

Supplement: Supplementary Materials — Figure S1: a flow chart for the treatment of MOG-Ab + ON. (a) The flow chart for the treatment of first-ever attack in MOG-Ab + ON. (b) The flow chart for the treatment of recurrent attacks in MOG-Ab + ON. Figure S2: the comparison of the thickness of the retinal nerve fiber layer of the patients. (a) The comparison thickness of retinal nerve fiber layer at the first-ever attack among different quadrants (center, superior, nasal, inferior, and temporal) in patients with MOG + AQP4-ON. (b) The comparisons of thickness of the retinal nerve fiber layer among patients with MOG + AQP4-ON and MOG-AQP4 + ON at different attacks. (c) The comparisons of thickness of the retinal nerve fiber layer of the patients with AQP4-Ab + ON at different attacks. [file 4951491.f1.zip › 4951491.f1/Figure S2-AQP4 -RNFL OF OCT-V1-20210811.pdf]
